# Time-interval for integration of stabilizing haptic and visual information in subjects balancing under static and dynamic conditions

**DOI:** 10.3389/fnsys.2014.00190

**Published:** 2014-10-06

**Authors:** Jean-Louis Honeine, Marco Schieppati

**Affiliations:** ^1^Department of Public Health, Experimental and Forensic Medicine, University of PaviaPavia, Italy; ^2^Centro Studi Attività Motorie (CSAM), Fondazione Salvatore Maugeri (IRCSS), Scientific Institute of PaviaPavia, Italy

**Keywords:** sensory integration, sensory reweighting, haptic, vision, equilibrium, quiet stance, dynamic balance

## Abstract

Maintaining equilibrium is basically a sensorimotor integration task. The central nervous system (CNS) continually and selectively weights and rapidly integrates sensory inputs from multiple sources, and coordinates multiple outputs. The weighting process is based on the availability and accuracy of afferent signals at a given instant, on the time-period required to process each input, and possibly on the plasticity of the relevant pathways. The likelihood that sensory inflow changes while balancing under static or dynamic conditions is high, because subjects can pass from a dark to a well-lit environment or from a tactile-guided stabilization to loss of haptic inflow. This review article presents recent data on the temporal events accompanying sensory transition, on which basic information is fragmentary. The processing time from sensory shift to reaching a new steady state includes the time to (a) subtract or integrate sensory inputs; (b) move from allocentric to egocentric reference or vice versa; and (c) adjust the calibration of motor activity in time and amplitude to the new sensory set. We present examples of processes of integration of posture-stabilizing information, and of the respective sensorimotor time-intervals while allowing or occluding vision or adding or subtracting tactile information. These intervals are short, in the order of 1–2 s for different postural conditions, modalities and deliberate or passive shift. They are just longer for haptic than visual shift, just shorter on withdrawal than on addition of stabilizing input, and on deliberate than unexpected mode. The delays are the shortest (for haptic shift) in blind subjects. Since automatic balance stabilization may be vulnerable to sensory-integration delays and to interference from concurrent cognitive tasks in patients with sensorimotor problems, insight into the processing time for balance control represents a critical step in the design of new balance- and locomotion training devices.

## Introduction

Maintaining balance involves complex sensorimotor transformations that continually integrate several sensory inputs and coordinate multiple motor outputs to muscles throughout the body (Ting, 2007). The control of quiet-standing posture consists in the maintenance of the center of mass (CoM) of the body within narrow limits. Also under dynamic balance conditions, like riding a platform periodically moving in the antero-posterior direction (Buchanan and Horak, 1999; Corna et al., 1999), the body requires accurate control of the CoM displacement within the range of the platform displacement. In both cases, the spatio-temporal activity of the agonist postural muscles (Schieppati et al., 1994, 1995; Tokuno et al., 2007; Kelly et al., 2012; Wright et al., 2012; Sozzi et al., 2013) is orchestrated by the central nervous system (CNS) based on one or multiple frames of reference (Peterka, 2002; Mergner et al., 2003; Schmid et al., 2007) upon which the body scheme is constructed (Haggard and Wolpert, 2005).

While keeping our body stable during the so-called “quiet stance” condition, feed-forward mechanisms are paramount in modulating the tonic activity in our antigravity extensor muscles and the correcting bursts in the antagonist muscles, which together control the displacement of the center of foot pressure (CoP; Morasso and Schieppati, 1999; Jacono et al., 2004; Bottaro et al., 2005, 2008; Loram et al., 2011; Vieira et al., 2012). In turn, these spatio-temporal patterns of activity rely on the knowledge of our orientation in space and of the relative position of our body segments during stance. This knowledge is built on multiple sensory inputs, which concur in the more or less accurate construction of the “internal model” of our body and of its relationship with the environment (van der Kooij and Peterka, 2011). The accuracy depends on the number and quality of the inflow from the various sensory modalities that have access to the centers integrating and using such information. Feedback obviously contributes to the instant-to-instant control of the stabilizing effort both by engaging reflex responses and by continuously updating the internal model (van Emmerik and van Wegen, 2002). Under steady-state conditions, the feedback contribution may be down-weighted by the brain (Peterka and Loughlin, 2004; Assländer and Peterka, 2014). Under dynamic but stabilized conditions, as when standing on a tilting platform and holding onto a still frame, the proprioceptive feedback from the legs is also down-weighted (Nardone et al., 1990; Schieppati and Nardone, 1991). During locomotion, alteration of the proprioceptive input from the leg muscle produces little effects on gait variables (Courtine et al., 2001). Thus, under predictable, steady-state conditions and tasks, be they static or dynamic, voluntary or produced in response to equilibrium perturbation, the excitability of the circuits ultimately called forth in the control of equilibrium may be tuned down. In general, sensory gating optimizes the execution of ongoing motor tasks (Clarac et al., 1992) by minimizing the effects on the motor command due to the inescapable delay from detection of the relevant information to its transmission to the neural generators of muscle activity (Suzuki et al., 2011). In this context, it is helpful to introduce an operative definition of postural set as it applies to both the control of body orientation in space and to the particular temporary level of excitability of the sensorimotor circuits underpinning the actual state of the body in its environment: “sensorimotor set is a state in which transmission parameters in various sensorimotor pathways have been adjusted to suit a particular task or context” (Prochazka, 1989). As such, the postural set, and in particular the neural circuits’ excitability to impending stimuli, is modifiable by the intention to change motor task and by the prediction of a change in the environment.

Stance stability depends on the availability and accuracy of the afferent stimuli that are integrated by the brain. The time-period whereby a sensory input is integrated and incorporated in the control of equilibrium is critical. For example, when the CoM is close to the border of its fixed support base (Schieppati et al., 1994), a handful of milliseconds can be enough to pass this limit and reach a condition that prevents any useful reaction. Any stabilizing information (e.g., vision) must therefore be rapidly integrated and rapidly produce corrective actions. Further, when we maintain the equilibrium during repeated and predictable perturbations of balance, anticipatory postural adjustments occur and in this context changes in visual conditions can quickly lead to appropriate modification in the anticipatory activities with appropriate changes in the balancing strategy (Corna et al., 1999; Schieppati et al., 2002).

The dependence of the control of human stance on sensory information has been the object of a great deal of investigations (Paulus et al., 1984; Day et al., 1993; Bronstein and Buckwell, 1997; Maurer et al., 2006; Guerraz and Bronstein, 2008). Much attention has been devoted to the central integration of afferent input from visual, somatosensory and vestibular receptors (Massion, 1994; Mergner and Rosemeier, 1998; Meyer et al., 2004; Borel et al., 2008). Changes in these sensory inputs lead the CNS to re-evaluate the respective contribution of the different sources of information for regulating posture (Oie et al., 2002; Peterka and Loughlin, 2004).

Ultimately, the more rapid the gain modulation on the insertion (or withdrawal) of a new stabilizing input, the shorter the time-period to reach the new appropriate postural set. Any information from the environment and from the body itself would concur in creating the better condition for the release of the postural muscle bursts apt to brake the displacement of the body’s CoM. It would be therefore appropriate if the CNS could integrate the stabilizing information within the shortest possible period of time.

The effects of changing sensory inflow during the performance of a coordinated complex motor task such as maintaining balance under quiet stance or dynamic conditions have received little attention so far (Rabin et al., 2006; Tax et al., 2013). The likelihood that sensory inflow changes during a complex movement is high, not only because of the obvious movement-related changes in proprioceptive input, but also because movement can imply passing from a dark to a lit environment, or from a stationary to a moving visual flow, or from a tactile-guided body displacement to an abrupt loss of such haptic-stabilized condition (Bove et al., 2006). The basic information for addressing these aspects of sensorimotor integration is fragmentary. Hence, the purpose of this review is to discuss sensory reweighting during static or dynamic balancing tasks. Particularly, the review focuses on the time-interval necessary for integration of balance stabilizing haptic or visual inputs, since this topic area is still relatively unexplored with most of the most relevant work having occurred in recent years.

## Visual information and stance stabilization

Vision affects both body sway during quiet stance (Schieppati et al., 1994; Nougier et al., 1998; Slobounov et al., 1998) and postural synergies when balancing on an oscillating platform (Buchanan and Horak, 1999; Corna et al., 1999; De Nunzio et al., 2005; Schmid et al., 2007). In a variety of situations, vision dominates over the proprioceptive input from a great number of postural muscles, the activity of which necessarily accompanies the standing task (Nardone et al., 1990; Bronstein and Buckwell, 1997; Redfern et al., 2001; van Emmerik and van Wegen, 2002; Hagura et al., 2007; Schmid et al., 2008; Carpenter et al., 2010; Murnaghan et al., 2011).

### Interaction of vision and proprioception

Regardless of the weight assigned to vision and proprioception by the brain, the interaction between the two sensory inputs may not be based on a simple algebraic sum, not least because of the different time-period necessary for the two inputs to access the brain, as shown by the different latency of their primary components in the cortical evoked potentials (Schieppati and Ducati, 1984; Bodis-Wollner, 1992; Shokur et al., 2013) or to reach consciousness (Barnett-Cowan and Harris, 2009). Further, the ultimate functional effects of either input or of their interaction over time relates to the particular current balance or movement constraints. For example, anticipatory muscle action preceding a predictable perturbation of quiet stance eyes-open is delayed by vibration of leg muscles (Mohapatra et al., 2012). On the other hand, relatively minor effects of muscle vibration are induced on the balancing behavior on a continuously oscillating platform in spite of vision being denied (De Nunzio et al., 2005). These findings open the issue of the effectiveness of leg muscle tendon vibration *per se* in modifying the control of balance, i.e., of a task strongly dependent on proprioception. This is not a matter of interest for this present review. Suffice it to mention here the intriguing finding that tendon vibration operates by triggering a vibration-frequency entrained discharge of the primary afferent fibers from the spindles (Hagbarth et al., 1973; Burke et al., 1976; Roll and Vedel, 1982; Matthews, 1988; Naito, 2002), while quiet stance relies mostly on the inflow of the secondary spindle afferent fibers (Schieppati and Nardone, 1995, 1999; Marque et al., 2001; Nardone and Schieppati, 2004; see also Pettorossi and Schieppati, under review).

Postural control provides an experimental context appropriate to highlight the interaction of multiple sensory inputs originating from different sensory systems (Hatzitaki et al., 2004). Body stability strongly depends on the non-linear aspects of the sensory fusion process and its temporal dynamics (Black and Nashner, 1984; Jeka et al., 2000; Horak and Hlavačka, 2002; Barnett-Cowan and Harris, 2009; Rowland and Stein, 2014). In turn, this depends to a large extent on the nature of the signals involved and their spatiotemporal relationship (Hlavačka et al., 1999). Experiments on the ability of young and elderly subjects to reconfigure their mode of stance control when submitted to successive reduced and augmented visual sensory conditions have shown a deficit in the operation of their central integrative mechanisms responsible for promptly modifying their postural control in the elderly (Teasdale et al., 1991). Young and elderly subjects’ body sway increased when occluding vision, while adding vision had a better effect on sway in young than the elderly, suggesting that elderly persons have a deficit in exploiting the stabilizing effect of vision (Jeka et al., 2010).

In a recent study, it was assumed that the sensory organization and the consequent postural set were influenced by the temporal relationship between visual and neck input (Bove et al., 2009), on the premise that re-weighting sensory inputs and re-shaping the postural reference frame must be a time-consuming process. In that paper, the authors investigated whether a given visual condition affects the postural response to neck vibration, and for how long does vision need to be absent prior to perturbation, before its stabilizing contribution be fully abolished. To this aim, the visual condition was time-manipulated to study its effects on the postural response to a balance-perturbing stimulus produced by neck muscle vibration. Notably, neck muscle vibration produces whole-body postural effects under both static and dynamic conditions (Lund, 1980; Roll et al., 1989; Lekhel et al., 1997; Ivanenko et al., 1999, 2000; Kavounoudias et al., 1999; Bove et al., 2001, 2002). The smallest postural response to vibration was observed when the eyes were open with respect to eyes-closed. This shows that vision is sufficient to significantly attenuate sway evoked by neck vibration. Conversely, the postural response to vibration eyes-closed that followed a period during which vision was allowed was significantly smaller than when vision was denied in the foreperiod. This indicated that the postural response to vibration is influenced not only by the visual condition during the administration of the vibratory stimulus, but also by the visual condition immediately preceding the vibration. A second finding was that, in the complete absence of visual references, the amplitude of the postural responses to vibration became progressively larger as a function of the repetition of the stimuli: in spite of the recovery to the initial position after each vibration pulse, the center of pressure moved forward to an increasingly larger extent during the successive neck vibration pulses, as if each vibration pulse found the postural control system progressively more susceptible to the abnormal proprioceptive input, when the absence of vision persisted. In a sense, the repeated proprioceptive perturbation eyes-closed progressively reinstated a heavy dependence of the postural control on proprioception or cancelled any postural reference constructed by visuo-somatosensory integration (Bottini et al., 2001). This sway-increasing phenomenon was not observed under eyes-open/eyes-closed condition, independently of the number of successive vibration pulses in the sequence. Clearly, presence of vision up to the beginning of vibration allows the CNS to define, and retain for a while, a stable postural reference able to cope with the threat represented by the abnormal proprioceptive inflow.

### Effects of vision on balancing behavior during a continuous predictable perturbation of stance

Standing upright quietly can hardly be considered a real balance challenge. Surprisingly, balance control under dynamic conditions (such as standing on a back-and-forth continuously translating platform) is not much more challenging either, at least as based on the observation that neither sensory nor motor impairment represent an unsustainable challenge to the elderly and patients with peripheral neuropathy or movement disorder (Nardone et al., 2000, 2006, 2007, 2008; Nardone and Schieppati, 2005, 2006). Certainly, subjects put in much more cognitive effort to sustain the performance level than under quiet stance (Beckley et al., 1991). Dynamic balancing behavior is an excellent experimental condition for assessing the role of vision in dynamic with respect to static equilibrium. There is indeed a remarkable difference in strategy depending on the availability of vision, whereby the balancing behavior shifts from that of a pendulum to an inverted-pendulum, passing from “head-fixed-in-space” behavior with eyes open to maximal body compliance to the perturbation with eyes closed (Corna et al., 1999). Incidentally, when blind subjects perform the task of balancing while riding a periodically moving platform, their strategy matches that of the sighted subjects performing eyes-closed (Schmid et al., 2007). This shows that long-term absence of visual information cannot be substituted by other sensory inputs (e.g., proprioception) for the selection of the balancing strategy in the control of equilibrium, in spite of the demonstrated cross-modal plasticity in blind subjects (Cohen et al., 1997; Kupers and Ptito, 2014). The findings point to the obligatory (though not unique, e.g., Panichi et al., 2011) role of vision in the processing and integration of other sensory inputs.

Schmid et al. (2008) investigated two competing hypotheses regarding the relationship between visual acuity and balance control strategy. One hypothesis referred to the existence of a threshold value of visual acuity as a turning point between the eyes-open and eyes-closed strategy. The other assumed that the change from eyes-open to eyes-closed balancing behavior is continuous and varies progressively with the worsening of the visual acuity. The findings showed that, in order to stabilize the head in space, visual information of the environment must be distinct. Reducing visual acuity leads to a graded modification of the “head-fixed-in-space” behavior. Thus, the body can produce a continuous mode of balancing patterns as a function of visual acuity. In a sense, this had already been shown by Paulus et al. (1984) for visual control of quiet stance. The findings suggest the notion that the central mechanisms for head and body stabilization operate through linear integration of the visual input with the general somesthetic feedback.

### Abrupt changes in vision during the continuous perturbation of balance

The previously mentioned studies have considered balancing behaviors to periodic balance-perturbing stimuli, under stationary sensory conditions (e.g., vision, reduced vision, or no-vision). They ignored relevant aspects of the postural behavior connected to transient sensory events. In subsequent studies, the time interval between the occurrence of a change in the sensory (visual) condition and the corresponding change in the motor behavior was investigated (De Nunzio and Schieppati, 2007). This interval includes the time to (a) integrate subtraction or addition of the sensory inputs; (b) shift from an allocentric reference (vision) to an egocentric reference (no-vision) or vice versa; and (c) adjust the calibration of the motor activity in time and amplitude to reach the best control appropriate to the new sensory set. A related question of adaptation to transient conditions had been previously addressed by Schweigart and Mergner (2008), who described a “sensory reweighting switch”, by which subjects change from a control that is referenced to the support to one that is referenced to space. Under optimal visual-acuity levels, on changing visual inflow during the trial (from eyes open to eyes closed or vice versa), the pattern of head and hip movement and of muscle activity turned into that appropriate for the new visual condition in a time-interval broadly ranging from about 1–2.5 s (De Nunzio et al., 2007). On the one hand, the findings indicate that subjects can rapidly adapt their balancing behavior to the new visual condition. On the other hand, the ample range of latencies across trials suggests that subjects refrained from releasing the new behavior when it was inappropriate, but rather released it at an appropriate time in the next platform translation cycle.

### Abrupt changes in vision during continuous perturbation of balance in patients with PD

Processing of sensory information and timing operations could be affected in Parkinson’s disease (PD) patients, who show abnormal calibration of postural responses (Schieppati and Nardone, 1991) or impaired flexibility of motor strategies (Horak et al., 1992). The capacity and swiftness to pass from a kinesthetic- to a vision-dependent behavior of these patients was investigated during the dynamic balancing task on the same continuously moving platform mentioned above. It turned out that both patients and normal subjects changed kinematics and EMG patterns to those appropriate for the new visual condition. However, PD patients were generally slower in changing their behavior under the eyes-closed to eyes-open condition (De Nunzio et al., 2007). These findings show abnormal temporal features in balancing strategy adaptation when shifting from kinesthetic only to kinesthetic plus visual reference in PD. The delay in the implementation of the vision-dependent behavior was unexpected, given the advantage vision is supposed to confer to motor performance in PD (Cooke et al., 1978). The delay on addition of vision in PD might be connected to an insufficient integration of a new sensory information in their body scheme, or to a delay in the implementation of the change in the appropriate balancing strategy (Bandini et al., 2001; Contreras-Vidal and Buch, 2003). This state might play a role in the instability of patients performing dynamic postural tasks under changing sensory conditions. Although static visual feedback reduces the walking patients’ reliance on kinesthetic feedback thereby favoring gait execution (Azulay et al., 1999; Lewis and Byblow, 2002), fast shifting to a new sensory reference may not be adequately exploited in everyday postural tasks. Venkatakrishnan et al. (2011) have suggested that *gradual* shifting of a new afferent input allows PD to better process the sensory input in a pointing movement.

### Measuring the delay between visual shift and implementation of the new balancing behavior in static condition

The great variability under the dynamic balancing conditions described above (Schieppati et al., 2002) does not allow to adequately address the issue of the sensori-motor processing time during sensory reweighting, owing to the complex motor task at hand. In a much simpler balancing condition, unaffected by the continuously variable kinesthetic inflow and relevant mechanical instability, the onset and time course of postural adjustments may be more clearly detected following abrupt sensory changes (from no-vision to vision or vice versa). Under these conditions, the stabilizing effect of vision is much less conspicuous than under more complex, balance challenging conditions (Buchanan and Horak, 1999; Corna et al., 1999; Ravaioli et al., 2005; Schmid et al., 2007); but it is definitely present (Paulus et al., 1984). The simple question was how long does it take for vision (eyes-closed to eyes-open) to stabilize posture, or how long does it take for the body to become less stable when vision is withdrawn?

The promptness of adaptation of stance control mechanisms was quantified by the latency at which body oscillation and postural muscle activity varied after a shift in visual condition. In a study aimed at estimating the promptness of adaptation to changes in visual conditions (Sozzi et al., 2011), volunteers stood on a force platform with feet parallel or in tandem. Shifts in visual condition were produced by electronic spectacles (LCD goggles that allowed or removed vision on receiving a TTL impulse). On allowing or occluding vision, decrements and increments in the CoP oscillation start occurring within about 2 s. These were preceded by appropriate changes in muscle activity, regardless of the visual-shift direction and the foot position during the standing task (feet parallel or in tandem). After the initial changes, EMG and CoP oscillations slowly reached the steady-state level corresponding to the new sensory condition within about 3 s. These figures were not dependent of the position of the feet, in spite of the overall larger sway under tandem condition, pointing to a constant duration of the sensorimotor integration process, hardly affected by the particular stance conditions at hand.

## Haptic information and stance stabilization

Very much as with vision, contact of the index finger with a stationary surface (Lederman and Klatzky, 2009) attenuates postural sway during quiet stance, even if the applied force itself (1 N) cannot provide mechanical stabilization. It has been proposed that slight changes in contact force at the fingertip give sensory cues about the direction of body sway (Holden et al., 1994; Jeka and Lackner, 1994; Jeka et al., 1997; Rabin et al., 1999, 2006; Krishnamoorthy et al., 2002; Kouzaki and Masani, 2008). Under steady state conditions, the effect of passive tactile cues during standing has been evaluated (Rogers et al., 2001) and the conclusion drawn that, if passive sensory input is available, the postural control process adapts to this input, better so the more cranial the point of application of the stimulus.

Sensory information from light fingertip touch (LFT) on a stationary surface can help in the case of loss of vestibular function (Lackner et al., 1999; Creath et al., 2002; Horak and Hlavačka, 2002). Therefore, LFT is relevant in the control of body orientation in space. Fingertip somatosensory input from an external reference provides spatial cues, which, akin to vision, facilitate the control of body equilibrium (see Wing et al., 2011). LFT has also been shown to suppress the destabilizing effect on posture induced by lower limb muscle vibration (Lackner et al., 2000). Of note, light touch contact between two individuals induced interpersonal stance symmetry (Johannsen et al., 2012). In other terms, the sway of the persons oscillating more would be reduced while the sway of the one oscillating less would be increased.

Stabilizing effects of LFT have been also described in normal subjects after lower-limb muscular fatigue (Vuillerme and Nougier, 2003), in healthy older adults (Tremblay et al., 2004; Baccini et al., 2007), in patients with peripheral neuropathy (Dickstein et al., 2001, 2003) or multiple sclerosis (Kanekar et al., 2013), and in patients with PD (Rabin et al., 2013) or bilateral vestibular loss (Lackner et al., 1999). Interestingly, LFT is able to relieve the perturbing effects of vibration-induced proprioceptive input from the neck, a segment central to postural control and orientation. LFT during neck vibration also attenuates vibration post-effects, further suggesting that its action is not merely mechanical (Bove et al., 2006). All these findings point to a paramount effect of the sensory inflow from light haptic touch on balance control.

### Haptic effects on reflex responses of postural muscles

Haptic information from a stable structure not only reduces the sway of the CoP during quiet stance, therefore of the CoM of the body, but also deeply modifies the excitability of the spinal proprioceptive reflexes that normally subserve the reaction to postural perturbations. By using a conditioning-test protocol, major effects of the haptic stabilization on reflex responses to postural perturbations have been observed (Nardone et al., 1990; Schieppati and Nardone, 1991). It was shown that stabilization of stance induced by holding onto a stable frame had a profound depressive action on the size of the medium-latency response to stretch of the postural leg muscles. This phenomenon was attributed to the change in the postural set. Interestingly, the reflex responses began to decrease about 200 ms before subjects touched the frame, but were not fully expressed until well after contact. The initial changes in amplitude of leg muscle responses are therefore not triggered by the go-signal or the contact with the frame itself, suggesting that the modulation is related at least in part to the central command to transition to a new stabilized postural set.

### Active and passive insertion or withdrawal of haptic information during stance

Thus, touch helps stabilize our standing body very much as vision does, but little is known about the time-interval necessary for the brain to process the haptic inflow (or its removal) and exploit the new information (or counteract its removal). Moreover, under conditions in which haptic information plays a stabilizing role, it would be interesting, on the basis of both basic and applied research data, to assess whether active touch or passive touch are equally effective (Chapman, 1994; Winter et al., 2008; Smith et al., 2009; Sciutti et al., 2010; Waszak et al., 2012), or significant differences exist, since our sensory systems are simultaneously activated as the result of our own actions and of changes in the external word (Von Holst and Mittelstaedt, 1950; Cullen, 2004). Active touch refers to the event where the subject would deliberately touch a surface, while passive touch refers to the event where contact with the surface would be established by external action without movement or anticipation of the stimulus by the subject.

**Figure 1:**
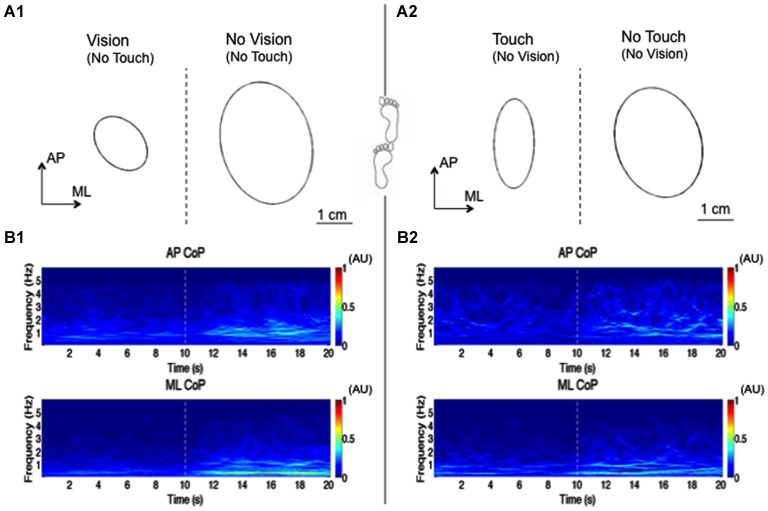
**Reweighting of visual or haptic information during tandem stance**. This figure shows an elaboration of the results obtained by Sozzi et al. (2011) in one subject standing upright under tandem-stance condition. In this experiment, the subjects’ visual sensory information was shifted from vision to no-vision (no touch), while haptic simulation was from touch to no-touch (blindfolded). The sensory shifts occurred at 10 s and were involuntary. The upper panel shows the ellipses of 95% confidence interval of CoP position (mean of 50 trials) during the vision/no-vision shift **(A1)** and touch/no-touch shift **(A2)**. Vision as well as haptic inflow decrease the area of the ellipse. The lower panel shows the “synchro-squeezed” (Daubechies et al., 2011) wavelet transform using a Morlet wavelet of AP CoP (upper traces) and ML CoP (lower traces) between 0.2 and 6 Hz. **(B1)** shows the transform during the vision/no-vision shift, **(B2)** during the touch/no-touch shift. The wavelet transform seen here is the mean of the transforms of 50 trials. The colors in the Figure represent the amplitude of the wavelet coefficient. Dark red represent the highest while dark blue is the lowest wavelets coefficient. Bins of 0.1 s have been chosen in order to better highlight the temporal changes in the coefficients after the sensory shift. Occluding vision or haptic information increases the wavelet coefficients in the frequencies ranging from 0.2 to 3 Hz, which indicates increase in the amplitude of the ML and AP oscillations. Higher frequency components were added up to the spectrum when sensory information was lost. The changes in the wavelets coefficient start increasing after a delay of approx. 1 s, to reach a stationary state in a few more seconds.

Sozzi et al. (2012) estimated the latency of onset and the time-course of the changes in postural control mode following addition or withdrawal of haptic information produced by touching (eyes-closed) with the tip of the index finger a strain-gauge instrumented touch-pad. Subjects were asked to actively touch the pad, or it was suddenly lowered or raised permitting to study the passive condition. The EMG of postural muscles during tandem stance was also recorded (in order to enhance muscle activity and body sway), to try to get as close as possible to the neural processing of the sensory information by eliminating the effect of the electromechanical delay. It had been shown previously that light touch stabilizes stance under both tandem stance and feet parallel 12 cm apart (Clapp and Wing, 1999). A summary representation of the modification in the medio-lateral and antero-posterior axes occurring around the instant of visual or haptic information shift is reported in Figure 1.

Muscle activity and sway adaptively decreased in amplitude on adding stabilizing haptic information. Across the subjects, the time-interval from the sensory shift to decrease in EMG and sway was ~0.5–2 s (Rabin et al., 2006). CoP followed the changes in tibialis anterior muscle EMG by ~0.2 s. Only slightly shorter intervals were observed following active sensory shifts (Pais-Vieira et al., 2013), in line with the conclusions by Winter et al. (2008) based on a stimulus timing-matching paradigm, who found no advantage on the perceived timing of an active over a passive touch. Latencies of EMG and postural changes were the shortest on removal of haptic information. Following the earliest detectable changes in amplitude, EMG and body sway reached the steady-state corresponding to the new sensory condition within ~1–3 s, under both active and passive tasks. Under control conditions, when subjects were asked to produce deliberate muscle activation in response to the sensory shift in a reaction-time mode, EMG bursts and CoP changes appeared at ~200 ms from the haptic shift, therefore much earlier than the adaptive postural changes seen during stance, signifying the operation of a different order of magnitude of the time scale of these events. Therefore, as much as for the visual information shifts mentioned above, changes in postural behavior require a finite amount of time from haptic shift. In particular, this delay from the sensory shift to the change in postural control mode was significantly longer for haptic than visual cues, the difference being much longer than that between the reaction times to the respective stimuli (Barnett-Cowan and Harris, 2009), indicating a modality-dependence and a heavier computational load for haptic information processing (Vuillerme et al., 2006; Tommerdahl et al., 2010; Bolton et al., 2011a).

The output of the sensory integration process seems to be issued to all relevant muscles. However, the latency of the change was shorter for the tibialis anterior muscle than soleus, likely because the latter rather plays a weight-bearing role (Schmid et al., 2011) while the former, along with peroneus longus, is responsible for providing medial-lateral stability in tandem-stance (Sozzi et al., 2013). Consistent with this role, the cortical projection to the tibialis anterior is stronger than to soleus (Valls-Solé et al., 1994). In this light, the shorter latency of the tibialis anterior changes would be an expression of a prominent supraspinal sensorimotor integration (Bolton et al., 2012) and fast cortical descending control. This finding would be in keeping with the proposals that the cerebral cortex plays a non-negligible role in the control of stance (Tokuno et al., 2009; Pasalar et al., 2010; Murnaghan et al., 2014).

It should be recalled here that the above delays are the result of a statistical estimation. Using statistics to document when a change occurs relies on assumptions and depends on the number of the cases upon which statistics is performed and the data variability, and cannot detect the “true” time at which a change at the CNS level occurs. Rather, the procedure will likely overestimate the true temporal locus of this change at the level of the CNS. Changes at the CNS level in response to visual or tactile inflow certainly occur before a value determined by using statistical tests (Soto-Faraco and Azañón, 2013; Heed and Azañón, 2014; Quinn et al., 2014). However, the same statistics and the same number of cases had been used in Sozzi et al. (2011) and Sozzi et al. (2012) when assessing both addition and withdrawal of information, and when comparing the time-periods to integration of haptic and visual addition (or withdrawal) in body stabilization, allowing a fair comparison to be made between the findings obtained with different sensory modalities and conditions. Admittedly, the “fuzziness” around when actual sensory events influence postural responses requires caution to be exercised to avoid precise claims on absolute times for when sensory signals play their role.

On reflection, one might wonder whether, in spite of all other things being equal, it was legitimate to compare the effect of the haptic sense from a minimal body surface (the tip of the index finger) with the visual information coming from a full binocular visual-field stimulation by the lighted and structured environment. Surprisingly, in spite of these disparities, the duration of the time-periods behind these sensory integrations and the extent of body-sway stabilization was remarkably consistent under both circumstances (Rogers et al., 2001), pointing to a sensory re-weighting phenomenon underpinning a change in reference frame rather than a central detailed analysis of the incoming information. Based on another analytical approach, Riley et al. (1997) had suggested an equivalent time-structure of the haptic and visual effects on the trajectory of the CoP.

### Haptic integration in blind subjects

Major reorganization of brain areas and reduced cross-modal interaction at the behavioral level follow congenital visual deprivation (Hötting and Röder, 2009; Fiehler and Rösler, 2010; Renier et al., 2014). Vision and touch rapidly lead to postural stabilization in sighted subjects, but is touch-induced stabilization more rapid in blind than in sighted subjects, owing to cross-modal reorganization of function in the blind? In people with impaired visual function, only minor differences in quiet stance control compared to sighted people have been reported (Rougier and Farenc, 2000). Jeka et al. (1996) found no differences between sighted and blind subjects on postural stability while using a cane, a task to which blind people are accustomed. Moreover, when exposed to sudden stance perturbation, the automatic postural responses of the blind are not substantially different from those of sighted persons (Nakata and Yabe, 2001). The same is true also for balancing while riding a periodically moving platform, where the balancing strategy of the blind subjects is similar to that of the sighted subjects performing eyes-closed (Schmid et al., 2007). The sensorimotor integration time of blind subjects should therefore be validly compared to that of sighted people under equal stance conditions. The aim of the Schieppati et al. (2014) study was to assess whether, in spite of known deficits in the processing speed of visual stimuli in the intact visual field of patients with visual system damage (Bola et al., 2013), blind subjects are more prompt than sighted subjects eyes-closed in reducing body sway in response to a haptic cue, based on their past experience and acquired skill in the use of their remaining senses (Pascual-Leone et al., 2005; Cattaneo et al., 2011).

Blind and sighted subjects, standing eyes closed with feet in tandem position, touched a pad with their index finger (LFT) and withdrew the finger from the pad in sequence. Steady-state body sway (with or without contact) did not differ between blind and sighted subjects. On adding the haptic stimulus, postural muscle activity and sway diminished in both groups, but at a significantly shorter latency (by about 0.5 s) in the blind (Schieppati et al., 2014). These data showed that blind are rapid in implementing adaptive postural modifications when granted an external haptic reference. Interestingly, the short delays appeared to be, at least in part, the consequence of a rapid learning process at the beginning of the series of trials, whereby the differences with respect to sighted subjects became obvious after some 10 task repetitions or so.

These findings show that fast processing of the stabilizing haptic spatial-orientation cues may be favored by neural plasticity in the blind, and add new information to the field of sensory-guided dynamic control of equilibrium in man. Under steady-state conditions, the balance control of blind subjects is not superior to that of sighted subjects eyes-closed. However, the former are considerably more rapid than the latter in implementing the appropriate modifications in postural set when confronted with a change in the relationship between body and environment. Coping with the haptic transient (rather than body stabilization *per se* under steady-state condition) seems to be favored by the loss of vision, perhaps through increased reliance on the sense of touch (Wong et al., 2011) and the enhanced functional connectivity between sensory and visual cortex (Ioannides et al., 2013; Ricciardi et al., 2014). The fact that the early-blind subjects showed a more prompt stabilization than late-blind subjects and that the latter were faster than in sighted subjects (Schieppati et al., 2014) suggests a progressive modification over time of the sensorimotor integration processes controlling body orientation in space, as part of their adaptation implying increased attention to non-visual events (Burton et al., 2014). Perhaps, the relatively lesser problems encountered by early-blind subjects in their activities of daily life compared to elderly, low-vision subjects (Chen et al., 2012) may be related to the early onset of plastic changes. In the view of these findings, protocols may be developed for enhancing both postural capacities and tactual object exploration and recognition (Tzovaras et al., 2004).

## What determines the length of the sensorimotor processing time?

What mechanisms contribute to the rapid decline in body sway following access to stabilizing haptic or visual sensory inflow? In stance control, under both static and dynamic conditions, we not only track with the CoP the random displacement of the CoM, but we bypass its instantaneous position, in the presumed direction along which it moves, in order to create the torque necessary for braking and reverting its displacement. Indeed, we act on the movement of the CoM, in order to constrain its displacement within a relatively narrow space. In doing so, we rely on the operation of complex processes, whereby ongoing sensory information may be able to inform about future states of instability in a predictive manner (Slobounov et al., 1997, 2009). This may not be dissimilar from the sheepdog task, promptly gathering and fetching moving animals to a pre-defined goal position (Vaughan et al., 1998). The narrower the surface of the ellipse within which the center of feet pressure—the flock—moves, the smaller the energy spent, and the more stable the CoM of the body. In the sheepdog model, the size of the overshoot can be greatly reduced by appropriately tuning the gain parameter—or increasing the dog’s anticipatory capacities.

Reducing the overshoot of the CoP with respect to the instantaneous position of the CoM to the extent sufficient for balancing with the minimal possible energy and computation costs would be achieved by increasing the gain of the system controlling the reciprocal positions of the CoM and of CoP, as if the sheepdog became “smarter” in controlling the flock. Changing the gain is likely operating by successive approximations, therefore time consuming, which might explain the relatively long delay of the onset of the changes in postural control mode and the slow time constant of the reduction in sway. Under different conditions (a computer-generated expanding visual field), likely requiring more complex processing than the simple abrupt change in haptic and visual information mentioned above, Jeka et al. (2008) measured the delay necessary for the nervous system to determine the most relevant sensory information for successful control of semi-tandem stance. Seconds from the change were necessary before a steady state was reached. Additionally, their data indicate a low speed for reweighting, when the visual scene motion was reduced, suggesting a temporal asymmetry (a slower process) whenever the change in the information does not threaten balance. Differences in the same sense (longer times to reach steady-state) have been also found on addition compared to withdrawal of stabilizing haptic and visual information, as mentioned above (Sozzi et al., 2012). Notably, under the condition of withdrawal of visual or haptic information, our nervous system could rely on its capacity for sustaining a working memory trace of recent information about the environment for guiding the reaction to postural perturbation (Bove et al., 2009; King et al., 2010; Cheng et al., 2012). Such a memory trace appears to explain our ability to guide targeted compensatory arm responses in the absence of online vision when a postural perturbation occurs (Cheng et al., 2013). However, this mechanism would not justify the *shorter* latencies of sway oscillation changes on withdrawal than addition of visual and haptic information under conditions of maintenance of unperturbed stance.

The timing of sensory modulation may differ when the task demands it and if the threat of an imminent fall increases the rate of gain modulation. For instance, threat of falling (Bolton et al., 2011b) or startling stimuli (Valls-Solé et al., 1999; Alibiglou and MacKinnon, 2012; Stevenson et al., 2014) can drive cortical motor responses faster than expected under normal conditions of voluntary control. Sensorimotor processes could as well be quickened when the task demands it. The slightly shorter latency of postural changes on *withdrawal* than addition of visual and haptic information would be affected by a similar event, since standing in tandem is more demanding in the absence of stabilizing information. Overall, one might note here that, however difficult the task of tandem standing, there is no urgent need to drive a rapid (and possibly metabolically costly) reweighting on the CNS, if a sufficient result can be managed with slower modulation.

## Conclusions and perspectives: brain augmentation and neuroprostheses

The likelihood that the inflow from different senses changes concurrently, or within a short time-interval, is non-negligible. This gives rise to new questions. Do concurrent changes in the “stabilizing” direction (e.g., from no-vision to vision and from no-touch to touch) summate and ultimately assure a “better”, more rapid performance? Are there differences when both changes occur in the opposite condition? Each stage of processing sensory information takes a certain amount of time, unique for each sensory modality (Barnett-Cowan and Harris, 2009): do these differences have an impact on the performance? Does the CNS, faced with a movement-balance integration problem, “select” one modality over the other in case of both changing? If so, are there “rules” for this selection? To what extent does the temporal order prevail over the modality? In this context, the expectation that the sensory condition(s) changes during the maintenance of a given (more or less critical) posture or in the preparation of a movement can play a role in the selection of the leading sensory information.

**Figure 2:**
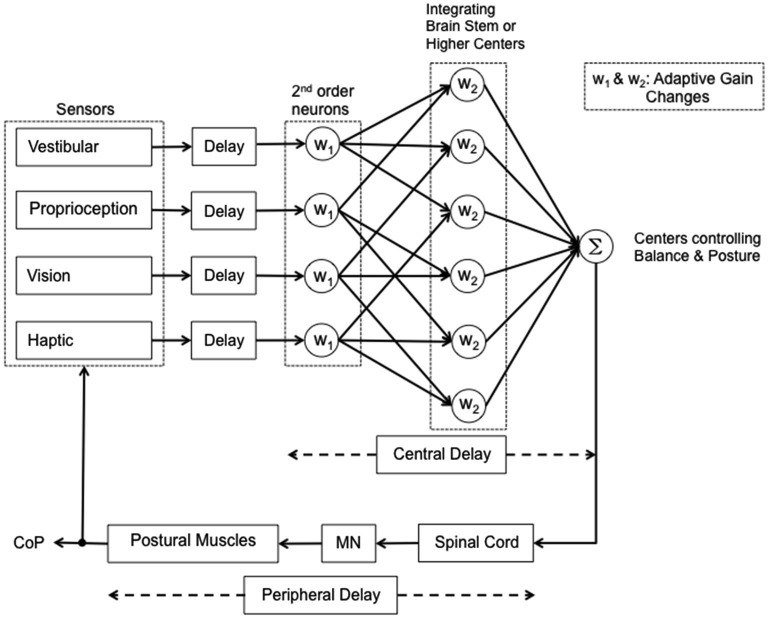
**Simplified scheme of the reweighting process during quiet stance**. Vestibular, proprioceptive, visual and haptic signals are coded by the peripheral receptors and reach the brain after a corresponding delay. The information is first processed in 2nd order neurons. The afferent information then diverges to higher integrating centers and may be then reweighted according to the availability and accuracy of the other sensory inputs and balance constraints. Then information converges again (Σ) in the centers responsible for the control of balance. Following a short delay the information is transferred to the spinal cord interneuronal circuitry that generates the appropriate spatio-temporal pattern of muscle activity. This implies activation of MN activity and relevant muscle force, the effect of which is measured as displacement of the center of pressure (CoP). Most likely, the main part of the interval between the shift in sensory condition and the change in CoP displacement (approx. 1–2 s) conditional to active or passive addition or withdrawal of sensory information) depends on the operation of the central mechanisms generating the adaptive gain changes.

These questions should be taken into account when considering problems of sensorimotor integration in elderly subjects or patients, and when designing simulation models of human balance. In perspective, aged persons (Nardone et al., 1995), Parkinsonian patients, and patients affected by peripheral neuropathies, and blind subjects (Bugnariu and Fung, 2007; Striem-Amit et al., 2012; Maidenbaum et al., 2014) represent examples of different conditions liable to affect the variable at hand, i.e., the sensori-motor processing time, due to progressive losses in function across multiple systems, including sensation, cognition, memory, motor control (Mahncke et al., 2006; De Nunzio et al., 2007; Nardone et al., 2007; Konczak et al., 2008, 2012; Schmid et al., 2008; Aman et al., 2014). A rough attempt at identifying possible steps of the sensorimotor integration process is reported in graphic form in Figure 2, where different reweighting coefficients are assumed for different modalities of posture-stabilizing information. Whether the coefficients also affect the delays should be checked by further investigations.

These mechanisms should have an impact on both basic knowledge and applied science: (1) The duration of the process of integration of a change in sensory information is an important variable in the field of sensory-motor coordination. It can be affected by various sensory and motor conditions, and be a marker of a normal state under a given condition. (2) Cognitive processing and integration of sensory inputs for balance require time, and attention influences this processing time, as well as sensory selection by facilitating specific sensory channels. Since performing a concurrent information-processing task may have an effect on the time delay, balance processes in older adults (Papegaaij et al., 2014) or sensory-impaired patients may be vulnerable to sensory-integration delays and to interference from concurrent cognitive tasks (Lacour et al., 2008). (3) Implementation of an appropriate time-lag between changes in a sensory modality, including its effects on balance, seems to represent an important aspect of the design of the control system for humanoid robots (Mahboobin et al., 2008, 2009; Peterka, 2009; Klein et al., 2011; Lebedev et al., 2011; O’Doherty et al., 2011; Rincon-Gonzalez et al., 2011; Demain et al., 2013). Biologically-inspired computational architectures, which are continuous in time and parallel in nature, do not map well onto conventional processors, which are discrete in time and serial in operation (Higgins, 2001). The findings briefly mentioned here would probably foster power- and space-efficient implementation technology. (4) “Rehabilitation robotics” is a new field of investigation between science and technology (Volpe et al., 2003; Casadio et al., 2008). Robots are being used to understand (Mergner, 2007; Mergner et al., 2009) and assist in maintaining balance and equilibrium (Forrester et al., 2014), or in helping movement practice following neurological injury (Krebs and Volpe, 2013), also providing insight into movement recovery. (5) Augmentation protocols of brain function offer enhancements for sensorimotor functions (this issue). For instance, appropriate patterns of vibratory stimulation to the dorsal axial trunk muscles easily reproduce functional medio-lateral oscillations of the standing body (De Nunzio et al., 2007) as well as enhance walking cadence and velocity in PD patients (De Nunzio et al., 2010). Moreover, evolved neuroprostheses employing functional neuromuscular stimulation (FNS) can restore basic standing function (Mushahwar et al., 2007; Braz et al., 2009; Capogrosso et al., 2013). Cochlear implants providing vestibular electrodes can enhance the function of the vestibulo-ocular reflex (Perez-Fornos et al., 2014).

Robots can haptically assess sensorimotor performance, administer training, and improve motor recovery. In addition to providing insight into motor control, robotic paradigms and sensory augmentation devices may eventually enhance motor learning and motor recovery beyond the levels possible with conventional training techniques (Steffin, 1997; Bach-y-Rita, 2004; Kärcher et al., 2012; Proulx et al., 2014; Wright, 2014). We hope that defining the sensorimotor processing time for balance can represent a small but critical step in the direction of building new, smarter balance and locomotion training devices.

## Conflict of interest statement

The authors declare that the research was conducted in the absence of any commercial or financial relationships that could be construed as a potential conflict of interest.
